# Preparation and Performance Evolution of Plasma Sprayed Abradable CuAl/PHB–NiAl Layered Seal Coatings

**DOI:** 10.3390/ma16010227

**Published:** 2022-12-27

**Authors:** Shuting Zhang, Wei Sun, Tong Liu, Jinhe Yang, Jianming Liu, Chao Wu, Peixuan Ouyang

**Affiliations:** 1 School of Mechanical and Material Engineering, North China University of Technology, Beijing 100144, China; 2 Beijing General Research Institute of Mining and Metallurgy, Beijing 100160, China

**Keywords:** plasma spray, abradable seal coating, microstructure, hardness, residual stress, oxidation resistance

## Abstract

In this study, a double-layered CuAl/PHB-NiAl seal coating was prepared on a GH4169 substrate by atmospheric plasma spraying. The evolution of the microstructure and mechanical properties of the coating under simulated working conditions was studied. The surface hardness of as-sprayed coating decreased with an increase in the temperature from 25 to 700 °C, decreasing from 90.42 HR15Y to 66.83 HR15Y. A CuO phase was formed in the coating and the oxidation weight gain rate increased with an increase in the temperature when the coating was constantly oxidized at 500~700 °C for 100 h. The hardness of metal matrix in the coating increased with the extension in the oxidation time at 600 °C, increasing from 120.8 HV_0.1_ to 143.02 HV_0.1_. The residual stress of the as-sprayed porous CuAl top-coating was less than that of the top-coating/bond-coating interface, and it is further relieved by about 15~20 MPa after heat treatment. The coating porosity first increased and then decreased when the oxidation time was 1000 h. The further ablation of PHB and the formation of oxide were concluded to be the main reasons for the evolution of porosity.

## 1. Introduction

Thermal spraying abradable seal coatings play an important role in gas turbine manufacturing, operation, and maintenance, and have been widely used in the engines field for more than 50 years [1,2]. As a typical sacrificial coating, the seal coating is applied to the inner surface of stator parts such as the casing. Based on the scraping effect, the clearance between the stator and rotor parts can be reduced to improve the sealing of the air path, thereby increasing the hydrodynamic pressure difference in the compressor, improving the overall efficiency of the engine, reducing energy consumption, and extending the service life of the whole machine [3,4,5].

Alloy material composition [6] and coating structure [7,8,9] in the abradable seal coatings are important factors affecting the service performance, service life and operation reliability of the coating. The seal coating is required to have good comprehensive properties which are affected by the severe and complex service conditions of the engine, mainly including hardness, bonding strength, thermal shock resistance, and abradability [10,11,12]. At present, much research on seal coatings for various systems has been reported. As a commonly used medium and a low-temperature seal coating system, AlSi-based and Ni-based seal coatings have always been the focus of research and improvement [13]. J.J. Tang et al. [14] studied the influence of microstructure refinement on the performance of AlSi/polyester seal coatings. The results showed that polyester refinement was conducive to improving the thermal stability and service performance of AlSi seal coatings. J. H. Cheng et al. [15] developed a finite element calculation model based on the microstructure of AlSi/polyester, which was further applied to study the changes in mechanical behavior caused by component changes, provided guidance for the microstructure design of AlSi/polyester coating materials. J. Ziegelheim et al. [16] explored the influence of flame spraying process parameters on the microstructure of a Ni/graphite seal coating, and pointed out that the velocity and deflection angle of flying particles are the key parameters affecting the porosity and nickel oxide content of the sprayed coating structure, respectively.

High temperature seal coating systems mostly use ceramic materials as the matrix skeleton phase, and the coating usually has higher hardness and temperature resistance. X.M. Sun et al. [17] prepared a yttria stabilized zirconia (YSZ) seal coating by using mixed solution precursor plasma spraying technology and improved nano-structured powder. They found that the oxidation resistance of this coating was improved compared with conventional spraying methods. T.T. Cheng et al. [18] compared and analyzed the thermal cycle performance of conventional YSZ high-temperature seal coating sprayed by air plasma and the modified coating added with whisker layer, and found that the thermal cycle life of the coating increased by 102.53% after adding whiskers. R. Ali et al. [19] prepared YSZ abradable seal coatings with different contents of MAX phase (Ti_3_AlC_2_) by atmospheric plasma spraying. The results showed that the hardness of the coating increased and the wear rate decreased with the increase in the content of MAX phase.

CuAl alloy has been widely used in the field of surface modification or component repair due to its excellent mechanical properties and corrosion resistance [20,21]. According to the Cu–Al binary phase diagram [22], this alloy system has good temperature resistance at around 600 °C and is one of the most promising material systems in medium-temperature seal coating. The residual stress and mechanical properties of the thermally sprayed coatings play a key role in its function and life [23]. In the field of thermal spraying, researchers mainly studied the residual stress and performance evolution of thermal barrier coatings under service conditions [24]. S.M. Zhao et al. [25] prepared 8YSZ/Eu coatings using atmospheric plasma spraying technology and studied the residual stress evolution during thermal cycling at 1300 °C. Z.Y. Wei et al. [26] developed a three-dimensional thermal barrier coating cracking model based on isothermal cycle test conditions and evaluated the dynamic growth behavior of cracks during the thermal cycle using the expansion finite element method. Based on the ultrasonic scanning results of the 8YSZ coating after thermal cycling, C. Deng et al. [27] established a physical and geometrical model for numerical simulation of three-dimensional cylindrical coatings, and simulated the stress distribution and evolution law of thermal growth oxides on the interface, sample center and edge by using the finite element method. Y.J. Cui et al. [28] used plasma spraying technology to deposit a dysprosium stabilized zirconia-based wear-resistant coating on a nickel base single crystal superalloy, and studied the microstructure evolution behavior of the ZrO_2_ based wear-resistant coating and matrix after thermal cycling at 1100 °C. However, there are few reports on the preparation and performance of seal coating with CuAl as the raw material powder component.

In order to further understand the relationship between the evolution of mechanical properties of CuAl seal coating and service time, it is of great significance to carry out research on coating properties under high temperature simulation conditions, which can provide guidance for improving the service life of seal coatings at high temperature. In addition, compared with many thermal spraying methods, such as line-arc deposition [29], suspended thermal spraying [30] and cold spraying [31], plasma spraying technology [32] has the advantages of high production efficiency, high energy utilization, large coating bonding strengths and wide range of sprayable materials. For the performance requirements of high bonding strength and uniform pore structure of the seal coating, it is most appropriate to use plasma spraying to deposit the CuAl seal coating.

To sum up, in this paper, Cu7Al (wt.%) and polyphenyl ester (PHB) were used as the alloy powder and the pore-formed powder for spraying, respectively. The CuAl/PHB–NiAl double-layer structure seal coating was prepared on a GH4169 substrate by atmospheric plasma spraying technology. The microstructure, residual stress distribution and conventional mechanical properties of the as-sprayed coating were analyzed. Additionally, the oxidation resistance and performance evolution of the coating were studied based on the simulated working conditions to understand the evolution mechanism of the CuAl seal coating. The research results are of great significance to the application of CuAl medium temperature seal coating systems.

## 2. Materials and Methods

### 2.1. Preparation of Coating Specimen

In this work, the raw material powder was prepared by the vacuum atomization process with an atomization pressure of 3.5 MPa. A CuAl/PHB primary composite powder with a particle size of 3~20 μm and a CuAl alloy powder with a particle size of 20~70 μm were mechanically mixed to prepare the final coating powder raw material. The mechanical mixing speed and time were 150 rpm and 2 h, respectively. The layered seal coating was prepared by atmospheric plasma spraying (GTV Verschleiss schutz GmbH, Luckenbach, Germany). A CuAl topcoat with a thickness of 0.6 mm was prepared on a GH4169 substrate with dimensions of Φ25 mm×5 mm by using a METCO F4 plasma spray torch. The nominal composition of the GH4169 alloy was Ni + 18Cr + 5Nb + 3Mo + 0.9Ti + 0.5Al + 0.03C (wt.%). The substrate surface was degreased, purified and roughened by sand blasting before spraying. A NiAl bond coat with a thickness of 0.1 mm was sprayed between the CuAl topcoat and the substrate in order to improve the bonding strength. The samples were air-cooled to room temperature after spraying. The coating powder composition and key spraying process parameters are shown in Table 1.

### 2.2. Constant Temperature Oxidation Test

CuAl-based seal coatings are a typical medium temperature seal coating system. The coatings were split into four groups and kept at a constant temperature of 500 °C, 620 °C, 650 °C and 700 °C for 100 h. A tubular resistance furnace (SAF Therm, Luoyang, China) was used to conduct constant temperature oxidation tests on the samples with a heating rate of 10 °C /min. The samples were weighed every 20 h with an electronic balance (YUEPING, Shanghai, China), and the oxidation resistance of the coating was judged according to the oxidation weight gain. In addition, in order to study the evolution of the microstructure and porosity of the coating after long-term high-temperature oxidation, static constant temperature oxidation experiments were carried out at 500 °C and 600 °C for 1000 h in a muffle furnace, and sampled at 5, 10, 50, 100, 500 and 1000 h.

### 2.3. Characterization

Scanning electron microscopy (SEM, Carl Zeiss, Oberkochen, Germany) was used to observe the microstructure of the CuAl abradable coatings, and an equipped energy dispersive spectrometer (EDS, Bruker, Bielerika, Massachusetts, USA) was used to analyze the microscopic composition of the oxidized coatings. Samples were prepared based on wire cutting, resin inlaying and conventional polishing. The average area fraction value of the black phase on the coating section in the SEM picture was calculated by Image J software (National Institutes of Health, Bethesda, Maryland, USA) based on six fields of view chosen randomly at 100× magnification. This represented the porosity of the coating. The phase of the coating after high-temperature oxidation was characterized by X-ray diffraction (XRD, Rigaku, Tokyo, Japan). The thermal expansion coefficient of the coating with dimensions of 2 mm × 2 mm × 10 mm was measured by a thermal expansion tester (NETZSCH, Selb, Germany). A surface Rockwell hardness tester (WOLPERT, Norwood, MO, USA) was used to test the hardness of the as-sprayed coating samples. The test temperature was 25~700 °C, and the selected hardness scale was HR15Y. The average value of five points for each sample was taken as the result. The Archimedes high-temperature hardness tester (ARCHIMEDES, London, UK) was used to test the hardness of the coatings after the thermal stability test. The test temperature was room temperature and 500~700 °C, and the selected hardness scale was HV_0.1_. The test was carried out in a vacuum environment. The loading force and the holding time were 100 gf and 10 s, respectively. Each sample was tested at five different positions at each temperature to ensure the accuracy of the test results.

An automatic gradient stress detection and analysis system (HUAWIN HAWKING, Jinan, China) was used to detect the non-uniform residual stress in the thickness direction of the coating sample. Firstly, the coating surface was polished to remove 0.05 mm as required by the patch, and then 20 layers were drilled from the top-coating surface with a step depth of 0.05 mm. The non-uniform principal stress was calculated by using the matching calculation software (HUAWIN HAWKING, Jinan, China).

## 3. Results and Discussion

### 3.1. Microstructure and Hardness of As-Sprayed Coatings

**Figure 1:**
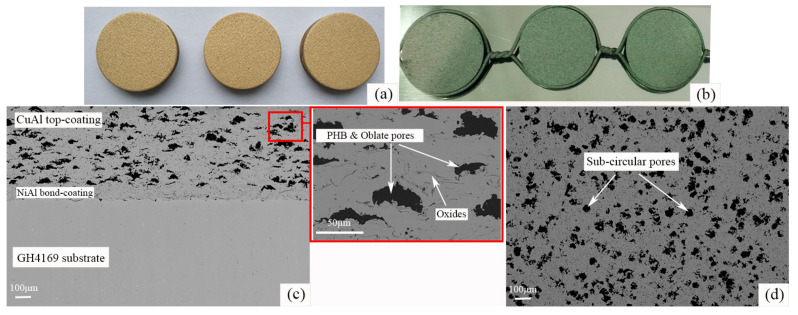
Surface macro morphologies of (**a**) the CuAl/PHB seal coating and (**b**) the NiAl coating; SEM images of (**c**) the cross-sectional microstructure and (**d**) the surface microstructure of the as-sprayed CuAl/PHB-NiAl abradable seal coating.

Figure 1a,b shows the macro morphologies of the as-sprayed CuAl/PHB and NiAl coatings, respectively. The surface of the CuAl surface layer was golden yellow, and the NiAl coating was silver-gray. The double-layer structure of CuAl/PHB–NiAl seal coating system is shown in the cross-sectional SEM image of the coating in Figure 1c. The spraying effect of the coating was good with continuous interfaces, and few defects were observed. By using the image measurement method, the total thickness of the coating was about 0.7 mm, which meets the sealing requirements of the inner wall of engine casings. During the spraying process, the fine particle-sized CuAl alloy formed a protective effect on the PHB, which reduced the burning loss and made the coating structure more uniform. The porosity of CuAl TC prepared by the optimized plasma spraying process and agglomerated composite CuAl/PHB powder was 15~20 vol.%. It can be seen from the enlarged view of Figure 1c that a typical porous honeycomb structure was formed. The black phase is PHB and pores, which make the coating abradable. The gray phase is the metal matrix mixed with filamentous interlayer oxides, which were formed by the stacking of powder droplets during thermal spraying. Figure 1d displays the surface SEM image of CuAl TC. The pore distribution of TC was uniform, and the pore diameter was mostly within 50~80 μm. Since the coating was sprayed by many powder droplets hitting the substrate or the surface of the deposited coating at a high speed based on mechanical bonding, it can be seen from the cross-sectional microstructure that the pores are oblate, while the pores are sub-circular shown in the coating surface SEM.

**Figure 2:**
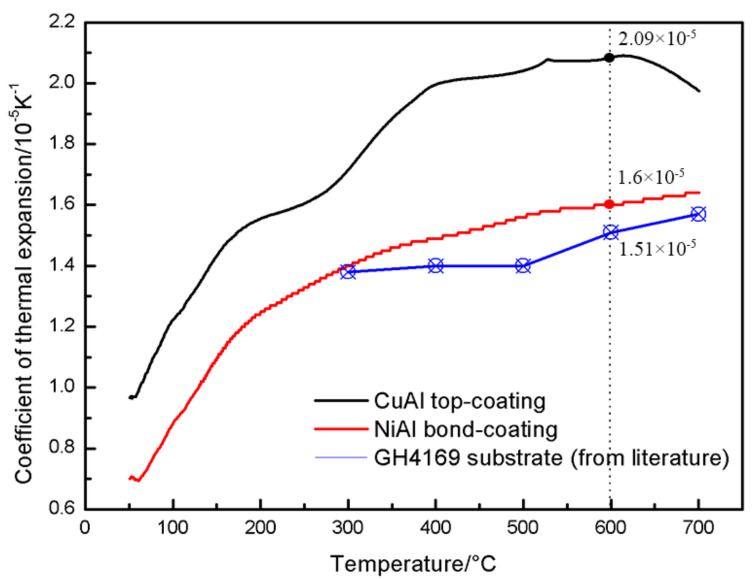
Thermal expansion coefficients of the thermally sprayed CuAl coating, NiAl coating and GH4169 substrate.

In addition, the intermediate bond-coating (BC) formed by thermal spraying of NiAl alloy powder has fine and dense structure, good uniformity, low oxygen content and porosity. The prepared NiAl BC can improve the bonding performance with the nickel-based superalloy. The coatings were fractured inside the coating after the tensile test, and its cohesive strength was 25~27 MPa, which fully meets the practical application requirements. Figure 2 shows the thermal expansion coefficient of the CuAl coating, the NiAl coating and the GH4169 substrate. The thermophysical properties of the NiAl coating at high temperature (the service temperature was about 600 °C) are between those of the GH4169 substrate [33] and CuAl TC, which can reduce the residual thermal stress inside and at the interface of the coating, to improve the thermal shock resistance and prolong the service life of the coating, further reflecting the importance of the NiAl BC.

**Figure 3:**
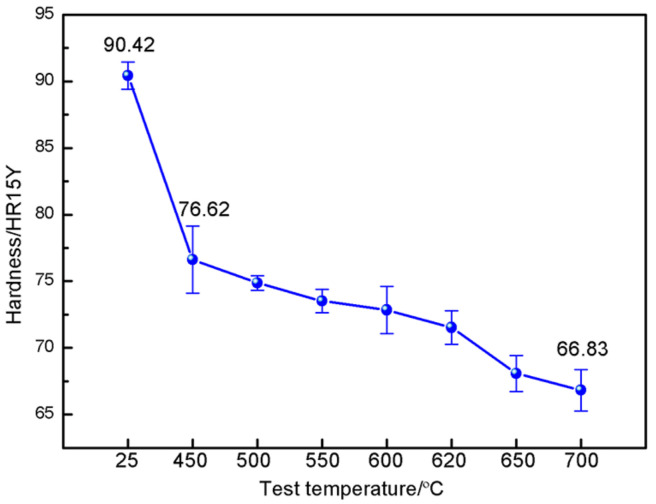
Effect of temperature on hardness on the as-sprayed CuAl seal coating.

The change curve of Rockwell hardness of the as-sprayed CuAl seal coating surface with test temperature is shown in Figure 3. The matrix phase of the CuAl alloy is the main source of hardness, and the room temperature hardness of the as-sprayed coating was 90.42 HR15Y. In general, the CuAl matrix phase softened with the increase in test temperature, and a decrease in hardness is conducive to scraping with the blades at service temperature. The surface hardness of the coating decreased from 76.62 HR15Y to 66.83 HR15Y when the test temperature was 450~700 °C, a decrease of 9.79 HR15Y.

### 3.2. Residual Stress Distribution of As-Sprayed Coatings

In this work, gradient drilling technology was used to measure the residual stress distribution of the coating, and the microstructure of the thin layer around the drilling-hole may change. However, as a destructive measurement method, the hole-drilling method has an advantage over others in calculation accuracy. In addition, metal-based coatings are not as easy to crack during the measurement compared with ceramic coatings [34], so it will not affect the residual stress state of the CuAl seal coating. The integral method is the most reliable analysis method for calculating the relaxation strain data of incremental drilling [35]. The residual stress in the thickness direction was evaluated based on the elastic modulus and Poisson’s ratio. The longitudinal, transverse and shear stresses obtained from the evaluation were converted to the maximum and minimum principal stresses by using Equations (1) and (2), respectively:

(1) $$\sigma_{max}=\frac{\sigma_{x}+\sigma_{y}}{2}+\sqrt{\left[{\left(\frac{\sigma_{x}-\sigma_{y}}{2}\right)}^{2}+\tau^{2}{}_{xy}\right]}$$

(2) $$\sigma_{min}=\frac{\sigma_{x}+\sigma_{y}}{2}-\sqrt{\left[{\left(\frac{\sigma_{x}-\sigma_{y}}{2}\right)}^{2}+\tau^{2}{}_{xy}\right]}$$

where *σ* and *τ* are the normal stress and shear stress, respectively. *x* and *y* are represent the transverse and longitudinal direction, respectively. All tensile stresses and compressive stresses are expressed as positive values and negative values, respectively.

**Figure 4:**
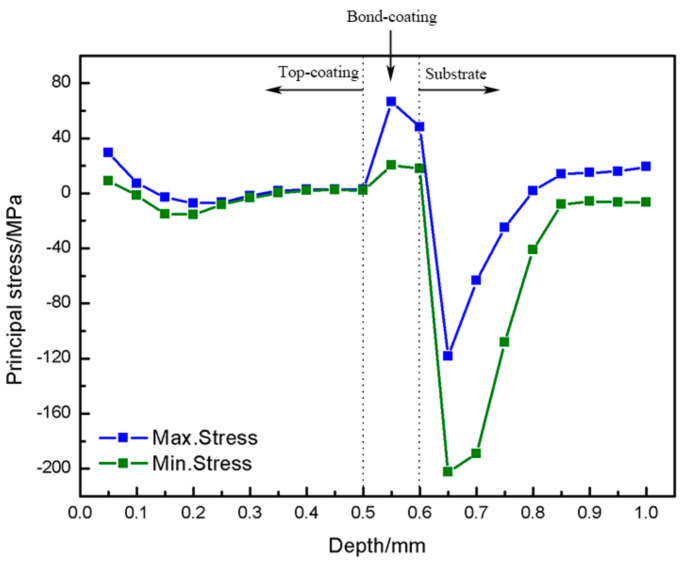
Residual stress distribution along the thickness direction of the as-sprayed CuAl seal coating.

In order to understand the characteristics of the stress distribution in the CuAl seal coating, the stress distribution along the 1 mm thickness direction of the as-sprayed coating surface is shown in Figure 4. It can be seen from the figure that the evolution trend in the maximum and minimum principal stresses of the coating/substrate system along the drilling direction is consistent. The stress magnitudes at the position of drilling of 0.05 mm, 0.5 mm and 0.6 mm represent the stress situation near the TC surface, TC/BC interface and the BC/substrate interface, respectively. The residual stress in the TC basically presented tensile stress when the drilling depth increased from 0.05 mm to 0.5 mm, and the maximum principal stress and minimum principal stress decreased from 29.44 MPa and 8.91 MPa to 2.71 MPa and 2.12 MPa, respectively. When drilling to the BC/substrate interface, the maximum principal stress and minimum principal stress in the 0.1 mm BC decreased from 66.38 MPa and 20.26 MPa to 47.96 MPa and 17.92 MPa, respectively.

Thermally sprayed coatings are usually composed of layered flakes or particles, including a few cracks and other defects. Residual stress combines the contribution of all coating components (including defects). The stress in various parts of the coating is non-uniform and the coating performance also changes in the thickness direction. Therefore, the seal coating performance is the comprehensive effect of various phases of the coating, as well as pores and microcracks. The residual thermal stress generated during thermal spraying is mainly thermal mismatch stress, which is caused by the temperature gradient and thermal expansion coefficient difference, and is closely related to the material properties and coating structure [36,37].

The initial temperature field of spraying changed sharply and the thermal expansion coefficient of dense NiAl BC was greater than the GH4169 substrate. Affected by the temperature gradient, the BC tends to stretch the substrate outward, resulting in tensile stress in the BC. The tensile stress accumulated and increased with the deposition of the BC. At the same time, the raw powder was instantly cooled into a solid deposition layer from the molten droplets during the spraying process, so a certain quenching tensile stress was also generated. In addition, the initial spray droplets hit the substrate surface at a high speed, producing a similar effect to the shot peening stress, resulting in a large compressive stress near the substrate surface.

When the thermal spraying deposition of CuAl sealing TC was continued, the temperature gradient was significantly reduced because the temperature of the deposited coating was still high. In addition, the CuAl composite powder was mixed with a certain amount of PHB, which formed many pores after sintering, and the thermal stress accumulated in the coating was released from around the pores instantaneously, resulting in a significant decrease in the residual stress of the TC. The test results show that the porous structure is helpful in improving the strain tolerance of the coating, alleviating the thermal stress and prolonging the service life of the coating.

### 3.3. Oxidation Resistance

**Figure 5:**
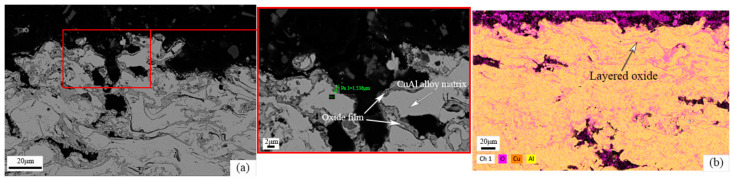
(**a**) Micromorphology of the coating after static oxidation at 500 °C for 100 h; (**b**) element distribution in micro-area.

**Figure 6:**
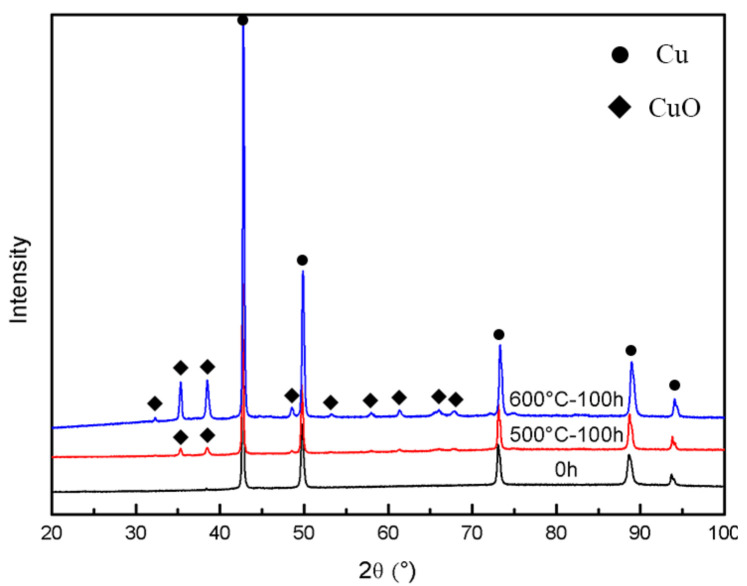
XRD patterns of the as-sprayed coatings after heat treatment at 500 °C and 600 °C for 100 h, respectively.

**Figure 7:**
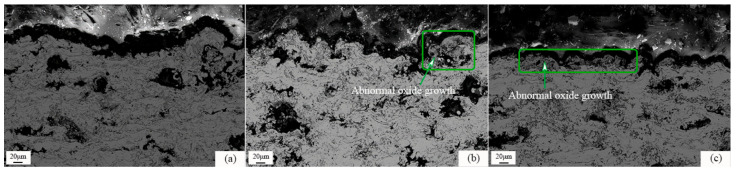
SEM images of the coatings after static oxidation at (**a**) 620 °C; (**b**) 650 °C; and (**c**) 700 °C for 100 h.

The microstructure and EDS photos of the CuAl seal coating after static oxidation at 500 °C for 100 h are shown in Figure 5. It can be seen from Figure 5a that the gray phase is the matrix and the dark gray phase is the oxide. The oxides are mostly generated around the pores. The thickness of the oxide film around the pores is about 1~2 μm, measured by the image method. XRD test results (as shown in Figure 6) show that the main oxidation product obtained inside the coating was CuO. In addition, according to the EDS results in Figure 5b, it can also be seen that the O element content in the interlayer pores, cracks and other defects inside the coating increased, combined with the further growth of the filiform interlayer oxides of the sprayed coating, resulting in an increase in the content of layered oxides. Therefore, the spraying process has an indirect impact on the oxidation resistance of the coating. Figure 7 shows the microstructure photos of the coating after static insulation at 620 °C, 650 °C and 700 °C for 100 h, respectively. It can be seen from the figure that the Al_2_O_3_ film on the coating surface is relatively continuous after oxidation at 620 °C, which helps to inhibit the diffusion of oxygen. However, the oxide film on the coating surface is irregular when the ambient temperature is 650 °C and 700 °C, and an abnormally grown oxide structure appears. The uniformity and density of the oxide film are poor, and it is difficult to prevent oxygen from entering the coating.

**Figure 8:**
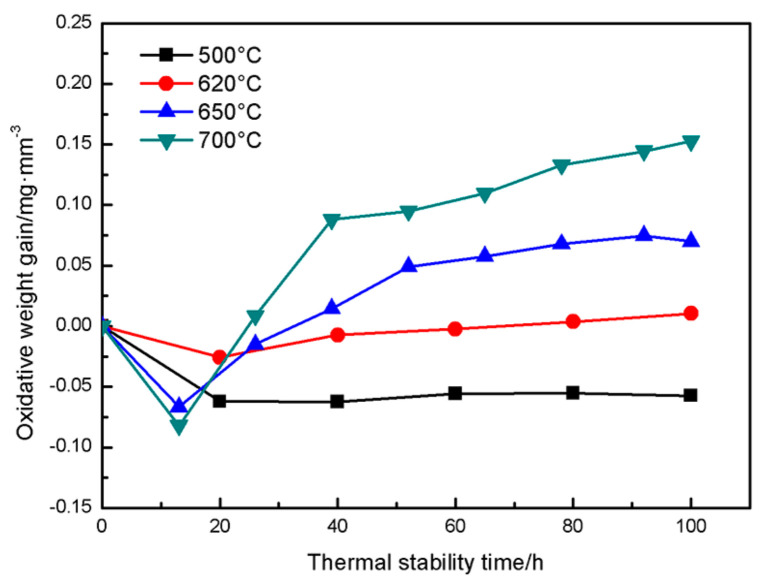
Effect of oxidation temperature and time on oxidation weight gain of the CuAl seal coatings.

In order to quantitatively analyze the oxidation resistance of coatings at different temperatures, Figure 8 shows the evolution of the coating oxidation weight gain with oxidation temperature and time. The average oxidation weight gain rates of the coating at 500 °C, 620 °C, 650 °C and 700 °C for 100 h was 0.6 × 10^−4^, 4.51 × 10^−4^, 11.5 × 10^−4^ and 19.5 × 10^−4^ mg/mm^3^·h, respectively. There are two phenomena of ablation of PHB and formation of oxide in the CuAl seal coating under high temperature service conditions. When the oxidation time is about 40 h, the ablation of PHB is dominant. At this time, it can be seen from Figure 6 that the weight gain of the coating is negative. As the thermal stability time is extended to 40 h, most of the PHB is ablated at this time, the oxide weight gain is dominant and the Al_2_O_3_ film formed on the coating surface also helps to inhibit the diffusion of O, so the oxidation rate of the coating is significantly reduced [38,39]. Combined with the oxidation weight gain and microstructure analysis, it can be concluded that the coating has good oxidation resistance below 620 °C, meeting the requirements of medium temperature seal coating systems.

### 3.4. Hardness and Residual Stress of As-Oxidized Coatings

**Figure 9:**
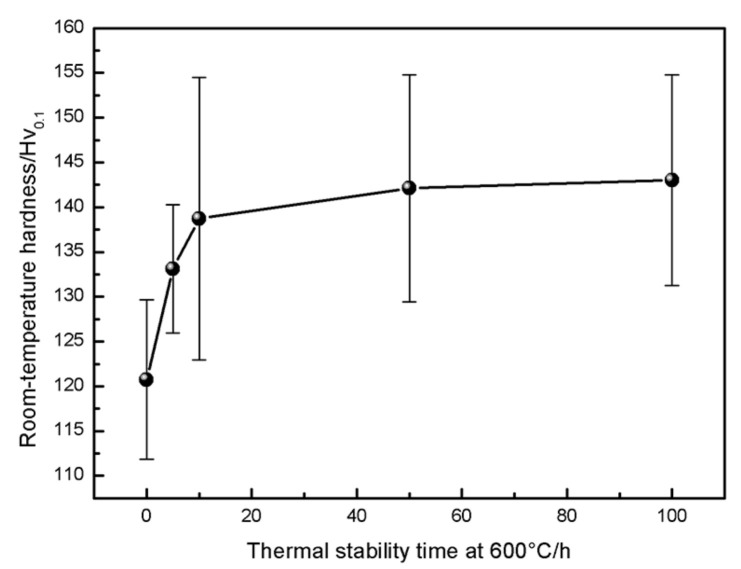
Effect of the thermal stabilization time on room temperature hardness of the CuAl seal coating.

In order to explore the influence of oxidation conditions on the hardness of the metal skeleton phase of the coating, the room and high temperature hardness of the coating were measured using the HV_0.1_ scale. Figure 9 shows the relationship between the thermal stability time at 600 °C and the room temperature hardness of the coating. The room temperature hardness of the as-sprayed coating (HV_0.1_, the same as below) was 120.8. The content of hard oxides in the coating gradually increases and segregates with the increase in constant oxidation time from 0~10 h. Therefore, the hardness of the coating increased with the increase in thermal stability time, increasing from 120.8 to 138.7, an increase of 17.9. The hardness of the coating keeps rising with the continuous increase in constant temperature oxidation time from 10~100 h, increasing from 139.7 to 143.02, an increase of 3.32. The increasing rate obviously slows down, which indicates that the coating structure tends to be stable, which is consistent with the trend in the oxidation weight gain experimental results.

**Figure 10:**
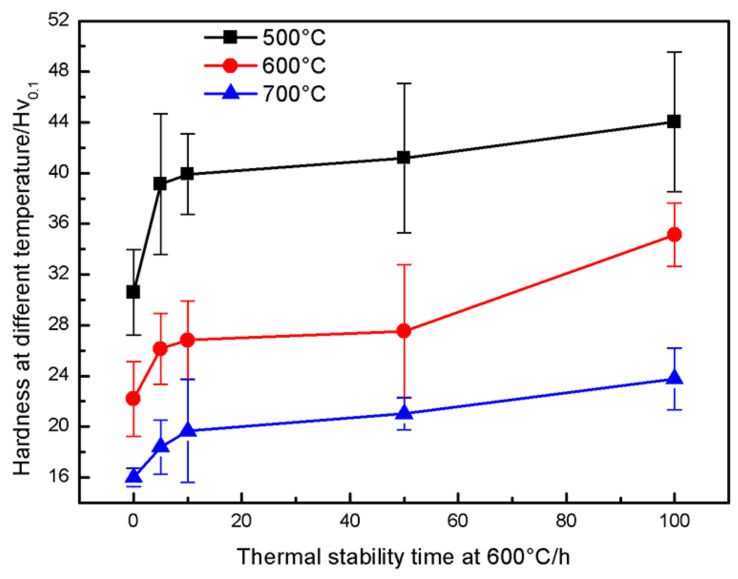
Effect of the thermal stabilization time on high temperature hardness of the CuAl seal coating.

Figure 10 shows the relationship between the thermal stability time at 600 °C and the high temperature hardness of the coating. On the one hand, the oxide content in the coating increases with the increase in thermal stability time, and its hardness gradually increases (as shown in Figure 9). On the other hand, the high temperature can soften the metal matrix of the coating [40]. The higher the test temperature, the lower the coating hardness. Compared with the hardness of the coating at room temperature, the hardness of the coating at high temperature decreased significantly. The hardness of the coating decreased from 30.6 to 16 when the test temperature was 500~700 °C, a reduction of 14.6. The hardness of the coating at 500 °C increased from 30.6 to 44 with the increase in the thermal stability time of 0~100 h, an increase of 13.4. The hardness of the coating at 600 °C increased from 22.2 to 35.1 with the increase in the thermal stability time of 0~100 h, an increase of 12.9. When the test temperature was 700 °C, the high-temperature softening was dominant, and the hardness growth trend slows down significantly due to the high temperature. The hardness of the coating at 700 °C increased from 16 to 23.8 with the increase in the thermal stability time from 0~100 h, an increase of 7.8.

**Figure 11:**
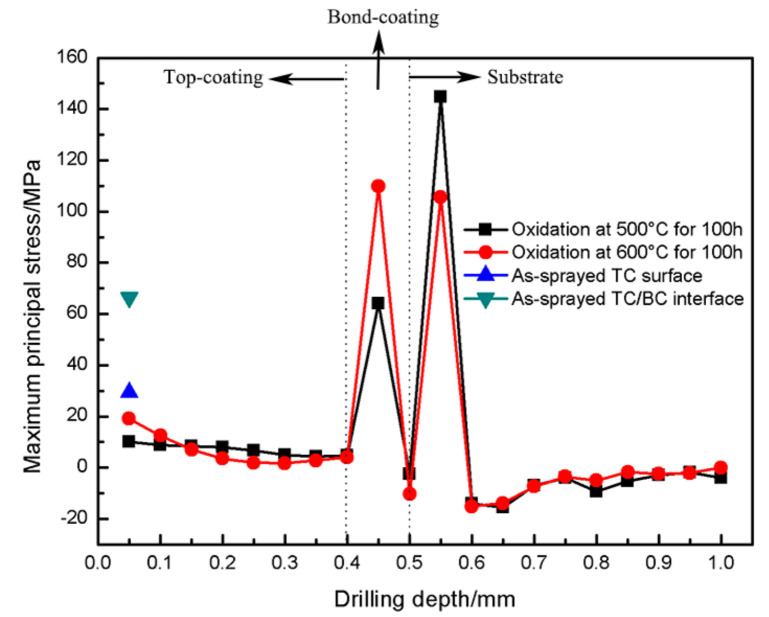
Residual stress distribution along the thickness direction of as-oxidized CuAl seal coating.

The samples were oxidized for 100 h at 500 °C and 600 °C, and the maximum principal stress distribution along the coating thickness is shown in Figure 11. The stress distribution of the two groups of samples is consistent. During the high-temperature oxidation of the coating, the residual stress in the CuAl TC will be redistributed and released around the pores. In addition, the process of oxide formation inside the coating will lead to a change in the coating volume, resulting in phase transformation stress. However, since the porosity of the TC is about 20 vol.%, the overall stress of the coating is still mainly affected by the porous structure. Therefore, it can be seen from Figure 11 that the oxidation experiment is helpful to eliminate the residual stress of porous TC. The residual stress of TC of two groups of samples at 500 °C and 600 °C was reduced from 29.44 MPa to 10 MPa and 19.09 MPa, respectively.

A certain thermal mismatch stresses will occur at the TC/BC interface and the BC/substrate interface during the heating and cooling processes of the oxidation experiment. In addition, during long oxidation times, oxides will be generated at defects such as pores and cracks in the NiAl BC, and the interlayer oxides generated during the stacking process of NiAl powder will also grow, resulting in large phase transformation stress. The phase transformation stress is usually tensile stress, but since the NiAl BC is a dense interlayer, the stress accumulation cannot be released. Therefore, compared to the as-sprayed coating, the internal residual stress of the NiAl BC after high-temperature oxidation showed little change, and there was still a higher tensile stress, which also caused the BC/substrate interface to change from the compressive stress of the as-sprayed coating to a larger tensile stress.

### 3.5. Effect of Long-Term Oxidation on Microstructure

**Figure 12:**
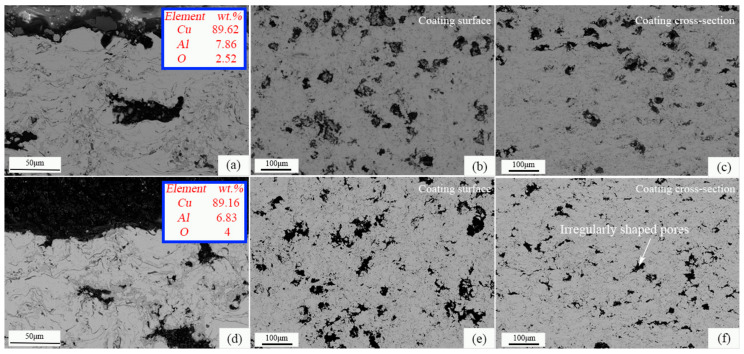
Microstructure and pore distribution of the CuAl seal coatings under constant temperature oxidation at 500 °C for (**a**–**c**) 500 h; (**d**–**f**) 1000 h.

**Figure 13:**
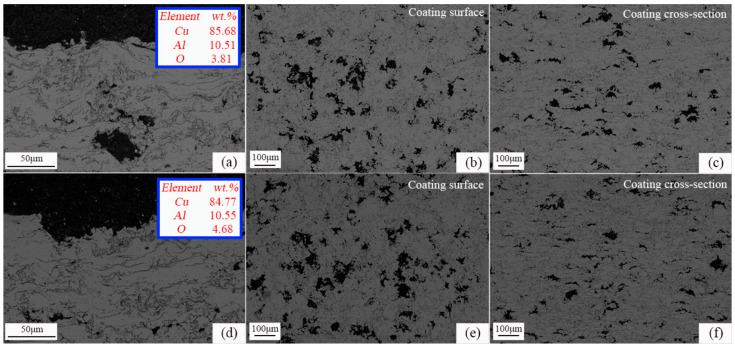
Microstructure and pore distribution of the CuAl seal coatings under constant temperature oxidation at 600 °C for (**a**–**c**) 500 h; (**d**–**f**) 1000 h.

Temperatures of 500 °C and 600 °C were selected to conduct 500 h and 1000 h long-term oxidation experiments based on results in 3.3. Figure 12 shows the images of the microstructures after being kept at a constant oxidation temperature of 500 °C for 500 h and 1000 h, respectively. It can be seen from Figure 12a,d that the content of O in the coating increased with the increase in oxidation time, indicating that the oxide grows further. The coating oxides mainly grow on the coating surface, around the pores and near the defects in the spray particle stack gap. When the oxidation temperature is 500 °C, the oxide film on the coating surface is uniform, with a thickness of about 1~2 μm. The main mechanism of oxidation resistance of the coating is the inhibition of the rapid diffusion of O into the coating by the surface oxide film. It can be seen from Figure 13a,d that when the oxidation temperature was 600 °C, the oxidation resistance of the coating was relatively reduced, and the uniformity of the surface oxide film becomes worse. The content of O corresponding to the coating at the same oxidation time increased, but the increment was not large, with an increase of 51.2% and 17%, respectively. It shows that the CuAl seal coating of this system has good microstructural stability at 600 °C compared with the same type of medium temperature seal coating.

**Figure 14:**
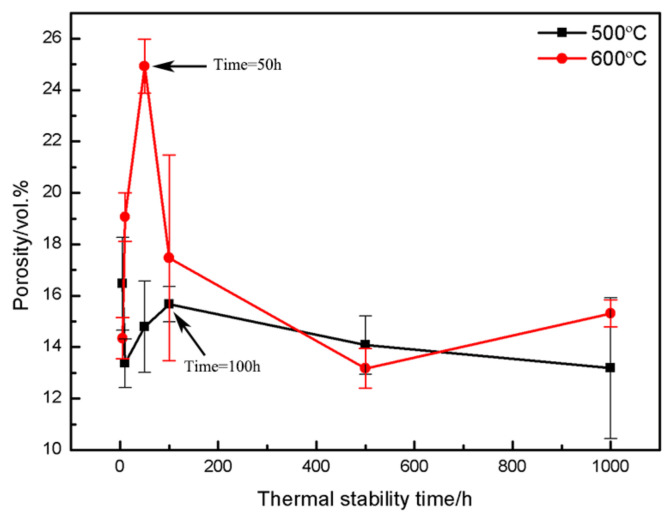
Effect of thermal stabilization time on porosity of the CuAl seal coatings at different oxidation temperatures.

Porosity is an important factor affecting the hardness and abradability of seal coatings. Figure 14 shows the effect of thermal stabilization time on the porosity of the coating at different temperatures. It can be seen from the surface and cross-sectional morphology of the coating in Figure 12 and Figure 13 that the pore shape changes with the increase in thermal stability time due to the further ablation of PHB and the formation of oxides during the heat treatment process. Especially when the thermal stability time is 1000 h, it can be seen from the image of the coating surface in Figure 13e that the pores evolve into irregular shapes, completely different from the sub-circular shape, in contrast with the as-sprayed coating, and the pore size tends to expand outward. It can be seen from the cross-sectional image of the coating in Figure 13f that the pore cross-section becomes more oblate, which is mainly due to the long oxidation time and the further growth of interlayer oxides. It can be seen from Figure 14 that there is an obvious evolution law between the coating porosity and thermal stability time under different oxidation temperatures. A large amount of PHB inside the coating is oxidized and ablated before the oxidation time is 50~100 h, resulting in an upward trend in porosity at this stage, which is consistent with Figure 8. With the further increase in oxidation time, the ablation phenomenon of PHB tends to be stable. Meanwhile, the oxide growth occupies a dominant position, which leads to the negative trend in coating porosity. After the long-term oxidation test, the coating porosity was stable at 15 vol.%. The results show that the CuAl seal coating has good microstructural stability under long-term high temperature service conditions, and it can be predicted that the coating can still maintain good abradability under high temperature conditions.

## 4. Conclusions

In this paper, a new component double-layer structure CuAl/PHB–NiAl seal coating was prepared on a GH4169 alloy substrate by atmospheric plasma spraying. The microstructure, porosity, surface hardness, tensile bonding strength, residual stress and oxidation resistance of the coating were studied, which showed good mechanical properties and thermal stability under as-sprayed and simulated working conditions. The research results can provide a reference for the application and development of CuAl-based seal coatings, but the mechanism of the influence of residual stress on the mechanical properties of the coating remains to be further studied. The main conclusions are as follows:

- The bonding between the two layers was good, and the microstructure of each layer was uniform. The surface hardness and cohesive strength of the coating were about 90 HR15Y and 25 MPa at room temperature, respectively, which meet the application requirements for medium temperature seal coatings of gas turbines. The surface hardness of the as-sprayed coating decreased with the increase in ambient temperature of 25~700 °C, decreasing from 90.42 HR15Y to 66.83 HR15Y, a decrease of 23.59 HR15Y. The porous structure of the as-sprayed TC can improve the strain tolerance of the coating and relieve the thermal stress. The surface stress was due to tensile stress and was less than that of the TC/BC interface, and the BC/substrate interface showed a large compressive stress.
- After 100 h of constant temperature oxidation at 500~700 °C, the coating metal matrix was oxidized and mainly formed a hard CuO phase. The oxidation rate was proportional to the temperature. At the same time, the hardness of the coating decreased with the increase in test temperature, and increased with the extension in oxidation time, but the growth rate gradually decreased. Constant temperature oxidation helps to release the stress of porous CuAl TC, and the surface stress of the TC was reduced by 10~20 MPa. Affected by the phase transformation stress, the BC/substrate interface changed from compressive stress, as present in the as-sprayed coating, to tensile stress.
- After constant temperature oxidation at 500 °C and 600 °C for 500 h and 1000 h, the microstructure was stable and the pores evolved into irregular shapes. The oxides were mostly concentrated on the surface of the coating and around the pores and were layered, induced by the spraying. The porosity of the coating first increased and then decreased with the extension in oxidation time, and finally stabilized at around 15 vol.%. It can be predicted that the high temperature abradability of the CuAl seal coating will be good. Further ablation of PHB and the formation of oxide are concluded as the main reasons for porosity evolution.

## Figures and Tables

**Table 1:** Material composition and process parameters of atmospheric plasma spraying.

Powders	Nominal Composition (wt.%)	Argon Flow Rate (L/min)	Hydrogen Flow Rate (L/min)	Feed Rate (g/min)	Electric Current (A)	Output Voltage (V)	Spray Power (kw)	Spray Distance (mm)
NiAl	Ni + 5Al	42	9.0	30–35	510	61	38	140
CuAl/PHB	Cu7Al + 3PHB	70	5.0	30–35	460	60	28	100

## Data Availability

Not applicable.
